# A 20 year experience in the management of non-tubal ectopic pregnancies in a tertiary hospital – a retrospective review

**DOI:** 10.1186/s12978-024-01838-6

**Published:** 2024-07-02

**Authors:** Theodora Hei Tung Lai, Jennifer Ka Yee Ko, Hung Yu Ernest Ng

**Affiliations:** Department of Obstetrics and Gynaecology, Queen Mary Hospital, School of Clinical Medicine, Li Ka Shing Faculty of Medicine, The University of Hong Kong, 6/F Professorial Block, 102 Pokfulam Road, Pokfulam, Hong Kong SAR China

**Keywords:** Ectopic pregnancy, Human chorionic gonadotrophin, Methotrexate

## Abstract

**Background:**

Non-tubal ectopic pregnancies account for < 10% of all ectopic pregnancies. Due to its rarity and wide variation in clinical practice, there is no guideline or consensus for its management. We reported our 20-year experience in the management of non-tubal ectopic pregnancies in a tertiary hospital.

**Methods:**

This is a retrospective review of all women admitted for non-tubal ectopic pregnancies from January 2003 to December 2022 in a tertiary hospital. Women with non-tubal ectopic pregnancies diagnosed by ultrasound or operation were included for analysis.

**Results:**

Within the study period, 180 women were diagnosed to have non-tubal ectopic pregnancies at a mean gestation of 6.8 weeks. 16.7% (30/180) were conceived via assisted reproduction. Medical treatment was the first-line management option for 81 women, of which 75 (92.1%) women received intralesional methotrexate administered under transvaginal ultrasound guidance. The success rate of intralesional methotrexate ranges from 76.5% to 92.3%. Intralesional methotrexate was successful even in cases with a positive fetal pulsation or with high human chorionic gonadotrophin levels up to 252605U/L. Twenty seven women were managed expectantly and 40 underwent surgery. Nine (11.1%), two (6.1%), and one (2.3%) women required surgery due to massive or recurrent bleeding following medical, expectant, or surgical treatment. Hysterotomy and uterine artery embolization were necessary to control bleeding in one Caesarean scar and one cervical pregnancy.

**Conclusions:**

Intralesional methotrexate is more effective than systemic methotrexate and should be considered as first line medical treatment for non-tubal ectopic pregnancies. It has a high success rate in the management of unruptured non-tubal ectopic pregnancies even in the presence of fetal pulsations or high human chorionic gonadotrophin levels, but patients may require a prolonged period of monitoring. Close surveillance and readily available surgery were required due to the risk of heavy post-procedural intra-abdominal bleeding.

## Introduction

Ectopic pregnancies most commonly occur in the ampullary region of the fallopian tube. Ectopic pregnancies located at other locations are rarer. Interstitial pregnancy refers to ectopic pregnancies in the intramural portion of the fallopian tube. Interstitial pregnancy accounts for 1–6.3% of all ectopic pregnancies [1]. Other ectopic pregnancies include cervical, Caesarean scar, ovarian, and abdominal pregnancies.

Non-tubal ectopic pregnancies account for < 10% of all ectopic pregnancies [2]. Non-tubal ectopic pregnancies can present late and be associated with massive hemoperitoneum if ruptured [3, 4]. While a new nomenclature has been suggested by the latest European Society of Human Reproduction and Embryology (ESHRE) guidelines [5], earlier cases, including those from our presenting cohort, were classified according to the old nomenclatures. Historically and in our study, we refer to non-tubal ectopic pregnancy to include both interstitial pregnancies and ectopic pregnancies implanting in sites other than the fallopian tubes [6, 7]. While interstitial pregnancies are strictly speaking located anatomically within the fallopian tube, they tend to present differently from ectopic pregnancies implanted in the other portions of the tube. Interstitial pregnancies can grow to larger gestations and cause heavier haemorrhage due to the more muscular walls and blood supply. The management of interstitial pregnancies also share more similarities to Caesarean scar pregnancies (CSP) or cervical pregnancies when compared to other tubal ectopic pregnancies. Interstitial pregnancies have therefore been included in this cohort, as with earlier studies [6, 7].

Due to its collective rarity, current recommendations are still based on results from observational studies or case series from specialized centres. There is yet to be a consensus on the best management option for such conditions [8–10].

The 2.5% mortality rate of non-tubal ectopic pregnancies was higher than those of tubal ectopic pregnancies at 0.14% [11]. Fortunately, significant morbidity and mortality resulting from non-tubal ectopic pregnancies are now less common, due to an earlier diagnosis with routine ultrasound examinations and adjunctive human chorionic gonadotrophin (hCG) measurements for women presenting early pregnancy complications. There has also been a shift from open to minimally invasive surgical approaches or the use of medical treatments such as methotrexate [12–15]. Treatment options generally depend on a multitude of factors, including the gestational age at diagnosis, whether rupture has occurred, the patient’s desire for future fertility, and the expertise available [2].

Overall, non-tubal ectopic pregnancies still pose a diagnostic and treatment challenge for gynaecologists. Maternal mortalities from non-tubal ectopic pregnancies are still happening due to misdiagnosis and delayed treatment [16]. We reported our experience in the outcome of management in women with non-tubal ectopic pregnancies at a tertiary hospital in Hong Kong over 20 years, with emphasis on interstitial, cervical and CSP. We previously reported our early experience in non-tubal ectopic pregnancies [17, 18]. Given the surge of cases in the recent 10 years, and the availability and shift of our approach to medical treatment for non-tubal ectopic pregnancies, an updated review of our experience is warranted.

## Materials and Methods

This was a retrospective review of all women admitted for non-tubal ectopic pregnancies from January 2003 to December 2022 at the Department of Obstetrics and Gynaecology, Queen Mary Hospital, Hong Kong. The centre is affiliated with the University of Hong Kong, with an annual gynaecological admission of about 9,000 women. The study was approved by the Institutional Review Board (IRB) of the University of Hong Kong-Hospital Authority Hong Kong West Cluster (UW 23–638). Patients’ consent was waived because of the retrospective nature of the study.

Women were identified from the hospital admission records, and the Early Pregnancy Assessment Clinic records. The information was cross-checked with the Hong Kong Clinical Data Analysis and Reporting System (CDARS) to ensure all women managed for non-tubal ectopic pregnancies within the study period were included. CDARS is a territory-wide health registry of attendances and admissions to all public hospitals managed by the Hong Kong Hospital Authority. Those with non-tubal ectopic pregnancies diagnosed by ultrasound or operation were included and their medical records were reviewed to confirm the diagnosis. Data regarding their demographics, presenting symptoms, ultrasound features, serum hCG levels, and management outcome (including operative records and histology results if applicable) were analysed.

The management of each woman was determined by her symptoms, gestation, viability of the pregnancy, hCG levels and her preference. A standard operating procedure is available as guidance to clinicians within the gynaecological unit attending women with non-tubal ectopic pregnancies to determine the treatment. Medical treatments were offered as the first line for most interstitial, Caesarean scar, and cervical pregnancies if clinically stable. Methotrexate was given either intramuscularly at 50 mg/m2 body surface area, or in 25–50 mg boluses into the gestational sac using a 19G single lumen ovum aspiration needle under transvaginal ultrasound guidance. Serum hCG levels were monitored on days 1, 4, and 7, then weekly thereafter. Expectant management was considered exclusively for asymptomatic women with decreasing hCG levels and non-viable non-tubal ectopic pregnancies. For both expectant and medical treatments, hCG levels were serially monitored until normalised to < 10 IU/L. Further interventions were considered if the patient developed worsening symptoms, or if the hCG plateaued, rose, or dropped by < 15% from day 4 to 7 after primary treatment. Regular ultrasound examinations were arranged once every 1–2 weeks after diagnosis, and subsequently, 2–3 monthly if the patient remained symptom free with a satisfactory hCG decline.

Surgical management, via laparoscopic route for interstitial pregnancies whenever feasible, was offered to those with ongoing bleeding, unstable haemodynamic status, or following unsuccessful conservative management as listed above. Women could opt for surgical management as the first line if they strongly wished to do so.

Treatment success was defined as the resolution of hCG to < 10 IU/L without the need for secondary treatment.

The reproductive outcomes were reviewed from the territory-wide health registry until December 2023.

Data was collected and analysed using SPSS Statistics (Version 26.0 Armonk, NY: IBM Corp). Data were expressed as mean and standard deviation (SD) for continuous data or n and percentage for categorical data. Differences according to demographic and gynaecologic data were analysed by the student’s t-test or Mann–Whitney test for continuous data, and chi-square test for categorical data. A *p*-value < 0.05 was considered statistically significant.

## Results

A total of 192 patients were initially identified from the medical records and the CDARS system. Upon review of the medical records, 9 cases were excluded as the diagnosis was revised to either intrauterine pregnancy or tubal ectopic pregnancy. 2 were further excluded as they defaulted our medical appointments before a formal diagnosis was reached. There was one additional case of second trimester Caesarean scar pregnancy managed with trans-abdominal intralesional methotrexate, which has already been reported separately [19].

**Fig. 1:**
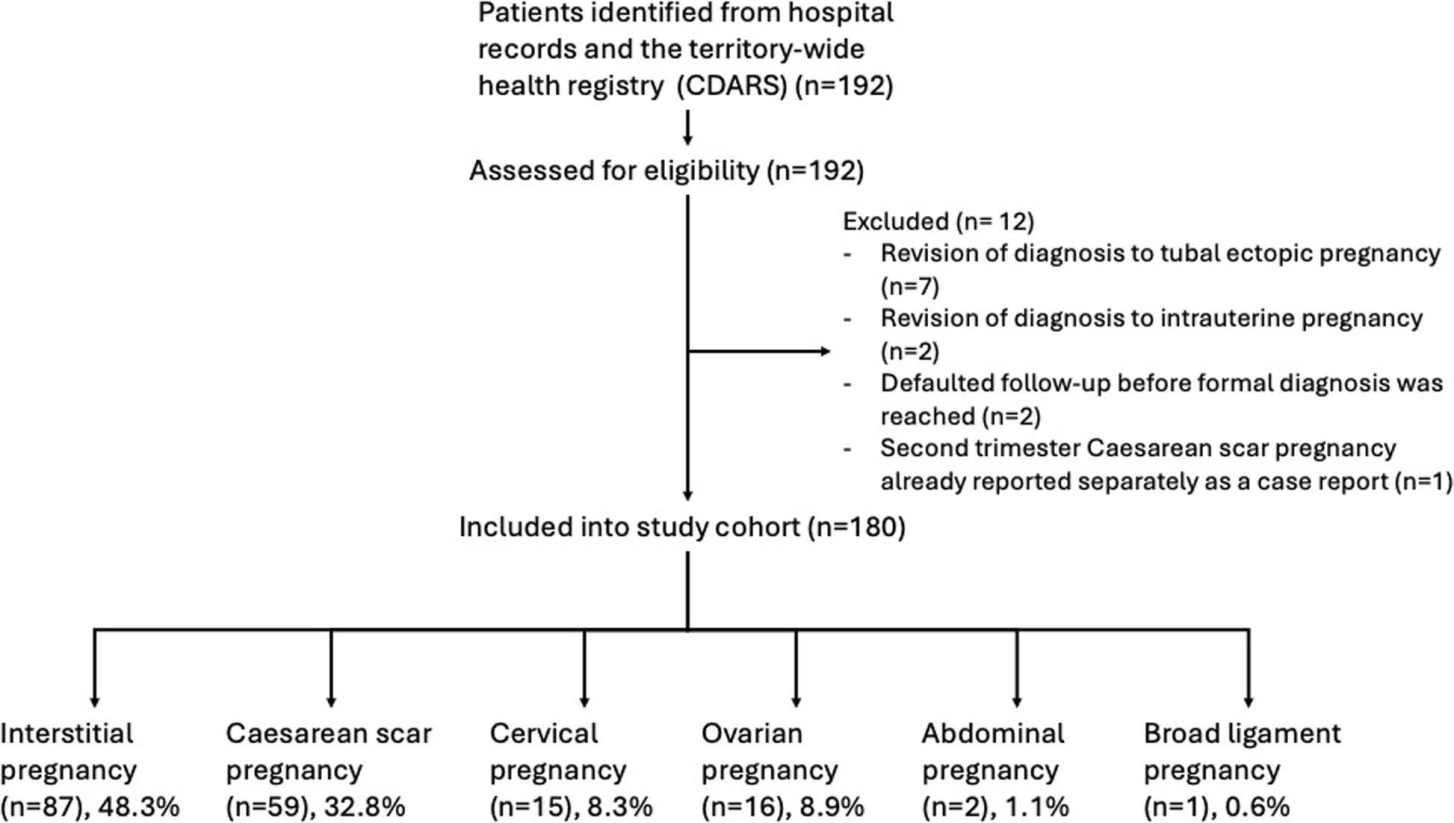
Flow diagram of study inclusion and types of non-tubal ectopic pregnancies

A total of 180 cases of non-tubal ectopic pregnancies were included, which accounted for 11.5% of the 1559 ectopic pregnancies diagnosed in the 20 years. Figure 1 shows the flow diagram for study inclusion and the breakdown of the types of non-tubal ectopic pregnancies.

**Fig. 2:**
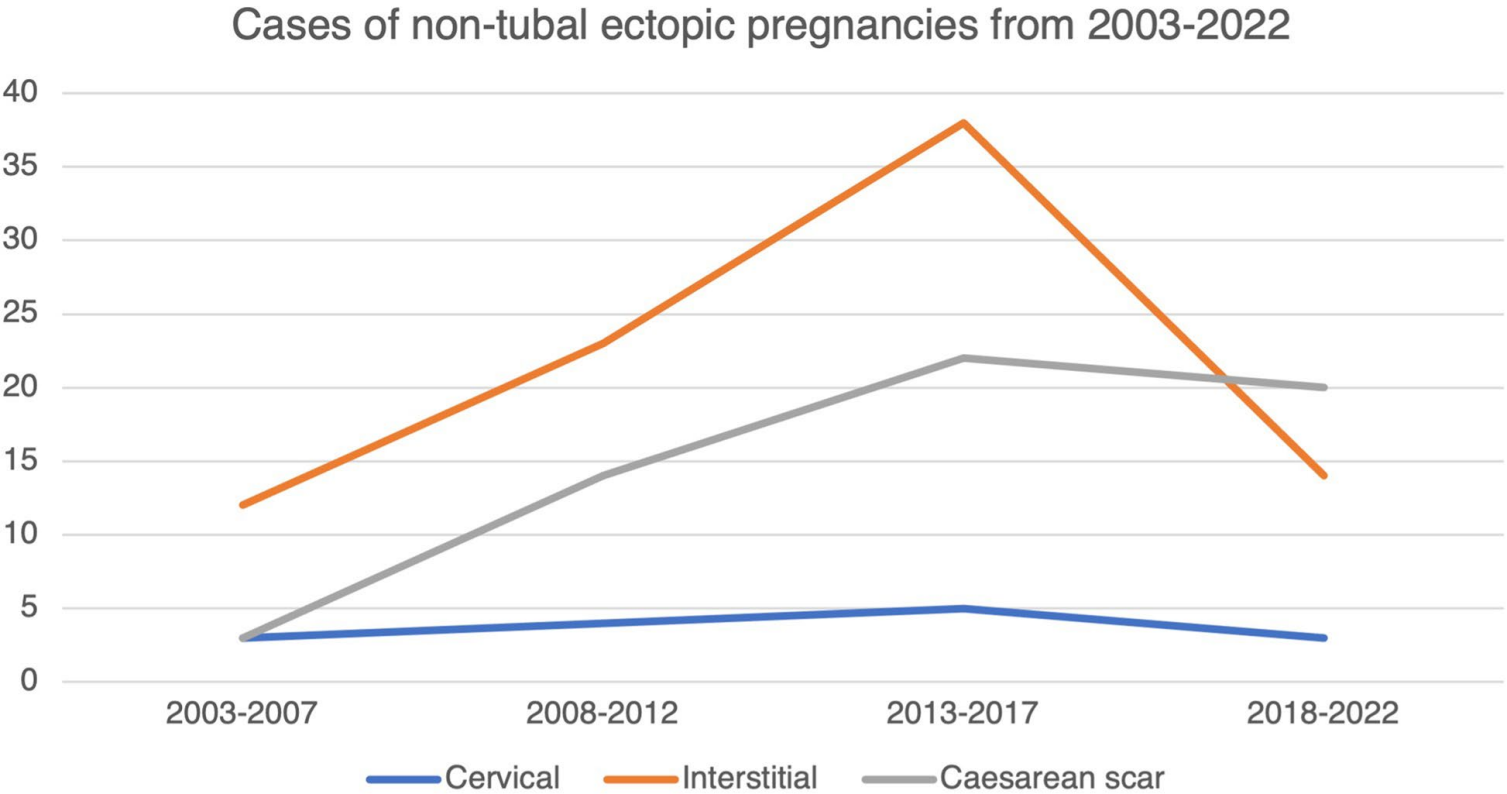
Cases of non-tubal ectopic pregnancies from 2003 to 2022

Overall, there was a rising trend in the proportion of non-tubal ectopic pregnancies (Fig. 2). Non-tubal ectopic pregnancies accounted for 8.1% (59/731) of all ectopic pregnancies in the first half of the cohort (2003–2012), compared to 12.3% (101/821) in the latter half of the cohort (2013–2022). The rise was mainly contributed by a surge of interstitial and CSP. In our cohort, up to 25% of women with interstitial pregnancies were conceived via assisted reproduction.

Table 1 shows their presenting clinical features. 87 women were diagnosed with interstitial pregnancy. 68 women were diagnosed accurately on ultrasound examination, while the remaining 19 women were diagnosed intraoperatively. The most common sonographic finding (70.6%, 48/68) was a gestational sac at the interstitial portion of the tube, with a mean sac diameter of 1.9 ± 1.5 cm. A fetal pole was present in 47.1% (32/68) and fetal heart pulsation was present in 32.4% (22/68) cases. Among those diagnosed intraoperatively, 11 women presented with hypovolaemic shock and received a provisional diagnosis of ruptured ectopic pregnancy before the emergency operation. The other 8 women were initially managed as tubal ectopic pregnancy, who presented with abdominal pain complicating early pregnancy with haemorrhagic shock. Ultrasonography showed an adnexal mass and an empty uterus, which was managed as presumed tubal ectopic pregnancy. Emergency diagnostic laparoscopy was therefore performed in view of clinically ruptured tubal pregnancy. However, these pregnancies were instead found to have implanted in the interstitial portion of the tube intraoperatively, thus their diagnosis was subsequently revised to interstitial tubal pregnancy after surgical management.

**Table 1:** Presentation of non-tubal ectopic pregnancies

Clinical features	Interstitial (*n* = 87)	Caesarean scar (*n* = 59)	Cervical (*n* = 15)
Shock or peritoneal signs	11 (12.6%)	1 (1.7%)	0 (0%)
Abdominal pain	37 (42.5%)	15 (25.4%)	3 (20.0%)
Vaginal bleeding	32 (36.8%)	52 (88.1%)	12 (80.0%)
Asymptomatic	25 (28.7%)	5 (8.5%)	4 (26.7%)

**Fig. 3:**
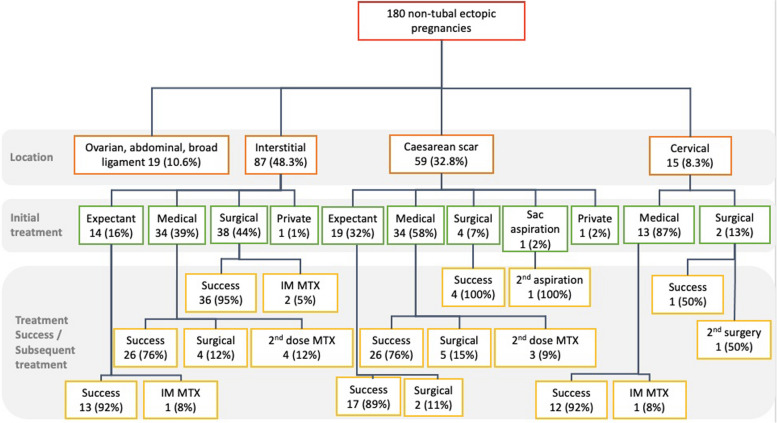
Flowchart of the types and overall management of the non-tubal ectopic pregnancies. IM: intramuscular. MTX: methotrexate

Figure 3 represents a flowchart of the overall management of our cohort. Table 2 shows the diagnosis, success of different primary treatment modalities, serum hCG, and ultrasound findings for the non-tubal ectopic pregnancies. Tables 3, 4, and 5 show the detailed mode of treatment for interstitial pregnancy, CSP, and cervical pregnancies respectively.

**Table 2:** Diagnosis, success of different primary treatment modalities, serum hCG and ultrasound for non-tubal ectopic pregnancies

Type	No of patients	Age (years)	Gestational weeks, mean (range)	ART, n (%)	Diagnosis by USG, n (%)	Initial treatment	No of patients, n (%)	Success rate of initial treatment	Initial hCG level IU/L (mean / median & range)	Time till hCG undetectable, weeks (median & range)	Time till USG normalised, weeks (median & range)
Interstitial	87	34.1	7.3 (4–16)	22 (25.3%)	68 (78.2%)	Expectant	14 (16.1%)	13 (92.9%)	20,205 / 7363 (307–62161)	10.7 (4–20)	15.7 (1–64)
						Medical	34 (39.1%)	26 (76.5%)	23,820 / 14,960 (1944–80,805)	8.9 (3–23)	33.8 (3–76)
						^a^Surgical	38 (43.7%)	36 (94.7%)	12,229 / 9473 (540–38584)	5.1 (1–10)	/
						FU private	1 (1.1%)	/	/	/	/
Caesarean scar	59	35.8	7.1 (4–13)	5 (8.5%)	59 (100%)	Expectant	19 (32.2%)	17 (89.5%)	16,512 / 2440 (86–130,297)	8.4 (2–20)	15.5 (3–28)
						Medical	34 (57.6%)	26 (76.5%)	51,820 / 34,305 (1963–141,607)	10.6 (5–22)	27.0 (4–84)
						Surgical	4 (6.8%)	4 (100%)	36,922 / 33,362 (16,886–64,078)	9 (5–13)	/
						Aspiration of sac	1 (1.7%)	0 (0%)	33,165	n/a	n/a
						FU private	1 (1.7%)	/	595	/	/
Cervical	15	35.4	6.1 (5–8)	3 (20%)	14 (93%)	Medical	13 (86.7%)	12 (92.3%)	48,441 / 22,472 (3127–252,605)	7.8 (3–17)	20.3 (3–39)
						Surgical	2 (13.3%)	1 (50.0%)	4636	43 (43)	/

^a^Includes 8 patients who were initially managed as presumed tubal ectopic pregnancy, where upon surgical treatment, was diagnosed to have interstitial pregnancy intraoperatively instead

**Table 3:** Mode of treatment for interstitial pregnancy

Mode of treatment	Number	Success (%)	Size of sac, mm (range)	Fetal pole evident n (%) CRL, range	Fetal cardiac activity evident n (%)	Initial hCG range (IU/L)		Secondary treatment / complications
						Success	Failed	
Expectant	14	13 (92.9%)	4–33	2 (14.3%) 4-6 mm	0 (0%)	307–51869	416	1 case – IM MTX for static hCG
Intramuscular MTX	3	3 (100%)	5–30	0 (0%)	0 (0%)	3485–5605	/	/
Intralesional MTX	30	22 (73.3%)	6–78	15 (50.0%) 2-12 mm	11 (36.7%)	2094–80805	9416–79,548	3 cases – 2nd dose intralesional MTX for rising hCG / persistent FH 1 case – laparoscopic cornual resection for rising hCG and abdominal pain 1 case – IM MTX for static hCG 2 cases – laparotomy for shock 1 case – laparoscopy for abd pain
Transvaginal aspiration of ectopic sac with KCl injection	1	1 (100%)	2.4	1 (100%) 7 mm	0 (0%)	n/a (heterotopic pregnancy)	/	/
Salpingotomy	12	11 (91.7%)	15–58	4 (33.3%) 6-23 mm	3 (25.0%)	331–37,721	9473	1 case—IM MTX for residual ectopic pregnancy
Salpingectomy / Cornual resection	26	25 (96.2%)	10–33	8 (30.8%) 3-60 mm	6 (23.1%)	541–38,584	12,348	1 case – IM MTX for static hCG
Further management in private	1	Unknown	/	/	/	Unknown	Unknown	/

*MTX* Methotrexate, *UAE* Uterine artery embolization

**Table 4:** Mode of treatment for scar pregnancy

Mode of treatment	Number	Success (%)	Size of sac, mm (range)	Fetal pole evident n (%) CRL, range	Fetal cardiac activity evident n (%)	Initial hCG range (IU/L)		Secondary treatment / complications
						Success	Failed	
Expectant	19	17 (89.5%)	6–30.6	8 (42.1%) 3–17.7 mm	1 (5.3%)	86–130,297	4400–31962	1 case – suction evacuation for heavy vaginal bleeding 1 case – suction evacuation and open hysterotomy for rupture and shock
Intramuscular MTX	4	1 (25.0%)	9.5–30	2 (50.0%) 7-26 mm	2 (50.0%)	3354	4402–90098	2 cases – suction evacuation for heavy vaginal bleeding 1 case – intralesional MTX for persistent fetal pulsation
Intralesional MTX	24	20 (83.3%)	7–30	20 (83.3%) 2-28 mm	19 (79.2%)	1963–115,115	15,099–109332	2 cases – 2nd dose intralesional MTX for persistent fetal pulsation / rising hCG 1 case – open hysterotomy for ruptured scar pregnancy 1 case – suction evacuation and UAE for vaginal bleeding and shock
Intralesional MTX + KCl	6	5 (83.3%)	20–60	6 (100%) 11–72.6 mm	6 (100%)	66,990–141607	139,653	1 case – suction evacuation for heavy vaginal bleeding
Laparotomy	1	1 (100%)	Not measured	1 (100%) 52 mm	1 (100%)	64,078	/	/
Suction evacuation	3	3 (100%)	11–23	0 (0%)	0 (0%)	16,886–46,136	/	/
Ultrasound-guided aspiration of sac	1	0 (100%)	9	0 (0%)	0 (0%)	/	33,165	Heterotopic pregnancy; transvaginal ultrasound-guided aspiration of sac done twice in view of persistent fetal pulsation
Further management in another centre	1	Unknown	/	/	/	Unknown	Unknown	/

*MTX* Methotrexate, *UAE* Uterine artery embolization

**Table 5:** Mode of treatment for cervical pregnancy

Mode of treatment	Number	Success (%)	Size of sac, mm (range)	Fetal pole evident n (%) CRL, range	Fetal cardiac activity evident n (%)	hCG range (IU/L)		Secondary treatment / complications
						Success	Failed	
Intralesional MTX	13	12 (92.3%)	7–51	4 (30.8%) 3-32 mm	2 (15.4%)	3718–252,605	9567	1 case—IM MTX for static hCG
Suction evacuation + balloon tamponade	2	1 (50.0%)	3.3	0 (0%)	0 (0%)	Not measured	4636	1 case—Cervical suturing + vasopressin + hysterotomy + UAE in view of recurrent heavy bleeding

*MTX* Methotrexate, *UAE* Uterine artery embolization

Medical treatment was the first line management in 50.3% (81/161), of which 92.6% (75/81) received intralesional methotrexate administered under transvaginal ultrasound guidance. The success rate of intralesional methotrexate ranged from 76.5% (26/34) for both CSP and interstitial pregnancies, to 92.3% (12/13) for cervical pregnancies. For women receiving medical treatment, the mean duration for normalisation of hCG and sonographic resolution was 9.1 ± 1.4 weeks and 27.0 ± 6.8 weeks (3–64 weeks).

11.1% (9/81), 6.1% (2/33), and 2.3% (1/44) of women required surgery due to haemorrhagic shock or suspected rupture following medical, expectant, or surgical treatment respectively. Hysterotomy and uterine artery embolization were necessary to control bleeding in three CSP and one cervical pregnancy.

Of the 59 women with CSP, the mean interval between their last Caesarean section and the index scar pregnancy was 4.6 ± 3.6 years. The most common indications for their antecedent Caesarean sections were done upon maternal request (37.3%, 22/59) and for previous Caesarean section (32.2, 19/59). The median overlying myometrial thickness was 1.9 mm (range 0.1-8 mm) at the time of sonographic diagnosis. Intralesional methotrexate was the most common primary treatment (50.8%, 30/59) with a success rate of 83.3% (25/30).

The reproductive outcomes of the cohort was reviewed until December 2023. One hundred fifty-four women expressed a desire for subsequent pregnancies, of which 45/154 women (29.2%) conceived following treatment of their ectopic pregnancies. Five pregnancies were results of assisted reproductive techniques. Two had previous cervical pregnancies, 10 had scar pregnancies, and 33 had interstitial pregnancies. The median interval from the sonographic resolution of their ectopic pregnancy to a subsequent pregnancy was 11 months (range 2–70 months). Two women still had a residual scar noted on ultrasound when the subsequent pregnancy was conceived 7 and 10 months later; both resulted in a full-term live birth. 82.2% (37/45) had live births, of which 59.4% (22/37) were delivered via Caesarean sections. All live births were full-term except 2 pregnancies – one woman had a twin pregnancy complicated with preterm labour at 36 weeks, while another had a Caesarean section for preterm prelabour rupture of membranes (PPROM) at 35 weeks. One woman with a previous scar pregnancy underwent a Caesarean section for placenta praevia and adherent placenta. Her operation was complicated with postpartum haemorrhage requiring uterine compression sutures.

Three women had a first trimester miscarriage. One woman with a prior scar pregnancy was admitted with pre-viable PPROM and spontaneous miscarriage at 20 weeks, which was complicated by retained placenta requiring suction evacuation. One woman underwent surgical termination of pregnancy. 3 women with prior interstitial pregnancies had a further tubal or interstitial pregnancy requiring surgical treatment.

## Discussion

We acknowledge the recent introduction of a new nomenclature for ectopic pregnancies. ESHRE has published new suggestions to classify ectopic pregnancies into uterine, extra-uterine, and rudimentary horn pregnancies. These are then further subdivided depending on their location, such as whether the uterine ectopic pregnancies are partial or complete. Standardized approaches to evaluate and measure ectopic pregnancies were also proposed [5]. Based on these updates, some of our ectopic pregnancies might have been reclassified. However, the diagnoses made in our cohort were based on recommended criteria from earlier guidelines, and we have decided to continue to report our findings using the older classification. We have now adopted the new nomenclature into our clinical practice. It is hopeful that future cohorts will be analysed based on the newer classifications. This would allow uniform reporting of ectopic pregnancies worldwide and easier comparison of results from different studies.

It is important to establish recommendations for women with non-tubal ectopic pregnancies to provide better counselling and management, especially given the dramatic rise in the number of women affected by non-tubal ectopic pregnancies over the past two decades. This was in part attributed to the increased use of assisted reproduction and the overall rising trend of Caesarean section [1, 20]. In our cohort, up to 25% of women with interstitial pregnancies were conceived via assisted reproduction, which is a well-known risk factor for ectopic pregnancies. Due to the disruption and scarring of the myometrium, the continued increase of Caesarean sections has caused a parallel increase in CSP and its complications [21]. Caesarean sections for maternal requests or social reasons increased from 9.0% to 15.1% between 2004 and 2014 in Hong Kong [22]. In the study centre, the rate of Caesarean sections also rose from 25.2% in 2003 to 31.6% in 2019. The recent reduction in the absolute number of ectopic pregnancies may be related to the drop in the overall pregnancy rates in the last five years, particularly during the COVID-19 pandemic.

The clinical features of non-tubal ectopic pregnancies are non-specific and could easily be confused with normally sited pregnancies. Clinicians should therefore be mindful of the possibility of ectopic pregnancies particularly if risk factors are present, given its increasing incidence.Ultrasonography confers a high degree of diagnostic accuracy even during early gestations, allowing the choice of conservative management including expectant or medical treatment. The use of three-dimensional ultrasound scanning can aid the diagnosis of non-tubal ectopic pregnancy. This technique was also utilised in the study unit for non-ampullary tubal ectopic pregnancy. For interstitial pregnancies, the intramural portion of the fallopian tube can be visualised more clearly with three-dimensional ultrasound [23]. It can also help differentiate it from other similar pathologies [24], allowing a more confident diagnosis.

However, despite the improvement in sonographic diagnosis, some interstitial pregnancies are initially misdiagnosed as tubal ectopic pregnancy. The diagnosis was finally made intraoperatively, when surgery was performed for ruptured ectopic pregnancy, presumed tubal in origin. Therefore, it is important to acknowledge that that for these interstitial pregnancies, surgery was the only possible management due to their clinical condition, and alternative options would be inappropriate in that clinical setting. However, they are still included within the cohort, as it is still important to review the outcomes of patients who received urgent surgical treatment.

Various management strategies have been reported for the management of women with non-tubal ectopic pregnancies. Whilst systemic methotrexate was considered the standard route of administration among women with ampullary tubal pregnancies [2], intralesional methotrexate has been gaining favour as a first-line option in women with non-tubal ectopic pregnancies. The intralesional route was adopted in over 90% of women in our unit. It showed overall success even amongst those with a high initial hCG level of up to 202605 IU/L or with a positive fetal pulsation. This suggests that the intralesional route could be considered even in situations when the systemic route would likely fail. With its availability, more women could become eligible for medical treatment, which is preferable to surgery for non-tubal ectopic pregnancies [25].

Previous studies found a high failure rate of systemic methotrexate of up to 45% in CSP [26], particularly in those with the presence of fetal poles. In our cohort, intralesional methotrexate was found to have a much improved success rate compared to systemic methotrexate in CSP (83% versus 25%), even when the former group had larger fetal poles with positive fetal pulsation present upon diagnosis. Our study, therefore, suggests a role of intralesional treatment for CSP, especially in the presence of fetal poles or pulsations.

There is currently no standard recommendation with regard to the drug regimen used for intralesional methotrexate. Dosages ranging from 10 to 75 mg have been reported [27, 28]. To date, no study has compared the effectiveness of different dosages. The dose and volume of medication injected were also limited by the size of the gestational sac. In the past, a standard dosage of methotrexate 25 mg (1 ml) was used by our unit, which was in line with earlier studies reporting this technique. It was however increasingly felt that a higher dosage of methotrexate might be more effective, particularly for ectopic pregnancies at more advanced gestations. We have recently doubled the methotrexate dosage to 50 mg (2 ml) since 2019 in selected women with larger gestational sacs. Systemic side effects or complications remained minimal among patients receiving a higher dose. Currently, it is too early to judge whether this increased dosage has led to an improved outcome. It would be worthwhile to evaluate the effects of the different dosages and to determine the optimum dosage in later studies.

The major disadvantage of medical treatment was the potential need for prolonged hCG and ultrasound monitoring following treatment. It is optimistic to note that the mean duration for normalisation of hCG was only 9 weeks, meaning that most women would not have to attend frequent medical reviews beyond 2–3 months of diagnosis. It is however important to counsel women to ensure realistic expectations before treatment, as the sonographic features of ectopic pregnancies can take an average 7 months to resolve.

It is also important to address the potential impact on women’s future fertility, given the relatively high proportion of women receiving assisted reproduction. There is currently no specific recommendation on the optimal pregnancy interval following treatment. However, of the 45 women who conceived after their non-tubal ectopic pregnancy within our cohort, 37 (82.2%) women achieved a live birth. The actual conception and live birth rates are likely even higher, given the possibility of unreported pregnancies that were managed elsewhere. Given the high live birth rates and low risk of recurrence of ectopic pregnancy, women should therefore be given reassurance with regards to their pregnancy outcomes, should they conceive post-treatment. In our study, two women conceived 7 and 10 months later with the presence of a residual scar on ultrasonogram. Both had uneventful deliveries. Uncomplicated pregnancies are therefore possible even before the sonographic resolution of their ectopic pregnancies, though no specific characteristics among the two women, given the small sample size.

The risk of recurrent ectopic pregnancy was about 6.7% (3/45) among those who conceived following treatment. All three women had prior interstitial pregnancies. Two underwent laparoscopic salpingotomy, while the remaining woman was managed conservatively. Their recurrent ectopic pregnancies were diagnosed early at 5–7 weeks gestation, which allowed adequate time for discussion and elective treatment before any complications had arisen. This highlighted the importance of an earlier medical review in any future pregnancies post-treatment.

The strength of our study included a relatively large cohort compared to other existing literature. All patients were managed in a tertiary centre with a standardized treatment protocol. The evolution of treatment options for non-tubal ectopic pregnancies over the past two decades could be evaluated.

There are certain limitations to the study. Though performed in a tertiary referral centre, all cases were recruited within a single centre study. Reproductive outcomes within our cohort may also be incomplete, as the data would not be available on the public health system if the patients opted for antenatal care or delivery in the private health system. The requirement of expertise for intralesional methotrexate may also limit the generalisability of our results. Nonetheless, this technique has been gaining popularity among various units both locally and internationally. Going forward, a territory-wide study would be useful to evaluate the management of non-tubal ectopic pregnancies.

## Conclusion

There is an overall rising trend of non-tubal ectopic pregnancies. Intralesional methotrexate should be considered as the first line medical treatment. It has a high success rate compared to systemic methotrexate in the management of unruptured non-tubal ectopic pregnancies. It can be used even in the presence of high hCG levels but could involve an extended period of monitoring. Close surveillance and readily available surgery were required given the risk of post-procedural bleeding or rupture.

## Data Availability

No datasets were generated or analysed during the current study.

## Abbreviations

CDARS: Clinical Data Analysis and Reporting System
CSP: Caesarean scar pregnancy
ESHRE: European Society of Human Reproduction and Embryology
hCG: Human chorionic gonadotrophin
PPROM: Preterm prelabour rupture of membranes
SD: Standard deviation
